# Astragalus Polysaccharides Target the Wnt/β-catenin Pathway to Suppress Malignant Behavior in Hepatocellular Carcinoma

**DOI:** 10.5152/tjg.2026.25188

**Published:** 2026-03-09

**Authors:** Li Liu, Wenyu He, Jiaoping Liu, Haiming Zhang

**Affiliations:** 1 Oncology Department of Integrated Traditional Chinese and Western Medicine, Huazhong University of Science and Technology, Wuhan Central Hospital affiliated to Tongji Medical College, Hubei, China; 2 Key Laboratory for Molecular Diagnosis of Hubei Province, The Central Hospital of Wuhan, Tongji Medical College, Huazhong University of Science and Technology, Hubei, China

**Keywords:** Astragalus polysaccharide, β-catenin pathway, epithelial-mesenchymal transition, hepatocellular carcinoma, Wnt

## Abstract

**Background/Aims:**

Despite some improvement in patient prognosis, hepatocellular carcinoma (HCC) remains a prevalent malignant tumor with disappointingly low overall survival rates. This study aims to investigate the inhibitory effects of astragalus polysaccharides (APs) on the malignant biological behavior of HCC and its underlying mechanisms, aiming to provide new strategies and theoretical foundations for HCC treatment.

**Materials and Methods:**

The inhibitory effects of APs on HCC were assessed using animal models and *in vitro *experiments. In animal models, different doses of APs were tested for their anti-tumor efficacy. *In vitro*, CCK-8 and Transwell assays assessed APs effects on HCCLM3 and HuH7 cell malignancy. Western blot analyzed Wnt/β-catenin pathway proteins and epithelial–mesenchymal transition (EMT) markers in APs-treated HCC cells. Rescue experiments confirmed APs-mediated inhibition of HCC behavior via Wnt/β-catenin signaling.

**Results:**

Animal experiments demonstrated that APs could effectively inhibit HCC development, with its inhibitory effect becoming stronger as the dose of APs increased. The *in vitro* data revealed that APs primarily inhibited the Wnt/β-catenin signaling pathway by downregulating β-catenin and p-GSK3β levels while increasing p-β-catenin levels, thereby inhibiting HCC malignancy. APs also regulated the expression of vimentin, N-cadherin, and E-cadherin—EMT markers. Moreover, *in vitro* rescue experiments indicated that APs induced apoptosis in HCC cells and inhibited their migration and invasion, which could be counteracted by a Wnt/β-catenin pathway activator.

**Conclusion:**

APs effectively curb the growth, spread, and invasiveness of HCC cells by targeting the Wnt/β-catenin pathway, thereby opening up new possibilities for HCC treatment.

**Main Points**

- Astragalus polysaccharides (APs) effectively curb the proliferation, migration, and invasion of hepatocellular carcinoma (HCC) cells.
- APs inhibit the Wnt/β-catenin pathway and regulate epithelial–mesenchymal transition (EMT) markers in HCC cells.
- The inhibitory effect of APs on the Wnt/β-catenin pathway contributes to suppression of HCC malignancy.

## Introduction

Hepatocellular carcinoma (HCC) constitutes the majority (80-90%) of primary liver cancers.^1^ The primary treatment options for HCC include surgical resection, liver transplantation, microwave ablation, systemic chemotherapy, immunologic treatments, chemoembolization (TACE), and transarterial radioembolization (TARE).^2^^,^^3^ While these methods have improved the prognosis of HCC patients to some extent, HCC remains a highly fatal disease, and targeted drugs for advanced HCC are still very limited.^4^^,^^5^ Consequently, developing innovative intervention strategies and identifying new therapeutic agents represent critical priorities in HCC early diagnosis and management. Traditional Chinese medicine has been proven to have unique efficacy in cancer treatment, which can offer benefits such as symptom relief, enhanced efficacy of chemotherapeutic agents, reduced toxicity of radiation and chemotherapy, and improved quality of life for patients.^6^

Astragalus polysaccharides (APs) are key bioactive compounds derived from Astragalus membranaceus, known for their diverse biological activities. These activities span anti-inflammatory, anti-aging, anti-radiation, anti-diabetic, anti-infection, and anti-tumor effects.^6^^,^^7^ APs can operate through multiple pathways, targeting various mechanisms to combat cancer. Specifically, APs can induce tumor cell apoptosis, regulate autophagy in tumor cells, inhibit tumor cell proliferation, and restore homeostasis in the tumor microenvironment.^6^ A prior study indicated that APs could enhance M1 macrophage polarization, promote dendritic cell maturation, and boost driven anti-tumor immunity, which collectively inhibit lung cancer cell growth.^8^ In another study, APs have been shown to inhibit prostate cancer cell growth via the miR-138-5p/SIRT1/SREBP1 signaling pathway.^9^ While these discoveries highlight the broad therapeutic potential of APs in cancer management, their mechanisms in HCC warrant further investigaton.

Polysaccharides have been reported to modulate pathways such as PI3K/AKT, MAPK, IGF-IR, Wnt/β-catenin, Fas/FasL, and TGF-β, which are associated with apoptosis, cell cycle arrest, and the inhibition of migration and invasion in gastric cancer cells.^10^ The Wnt/β-catenin pathway, or canonical Wnt pathway, is a central regulator of liver homeostasis and tumor development.^11^ This pathway is highly conserved across species and is activated when extracellular Wnt ligands bind to cell membrane receptors, typically via autocrine or paracrine signaling.^12^ Once activated, this pathway stabilizes β-catenin, allowing it to accumulate in the cytoplasm and translocate to the nucleus, where it activates genes involved in cell proliferation, survival, differentiation, and migration.^12^ In HCC, the Wnt/β-catenin pathway is often aberrantly activated, driving key oncogenic processes such as cell proliferation, apoptosis resistance, migration, invasion, and drug resistance.^13^^-^^15^ Research by Leung et al.^16^ revealed that activation of this pathway can enhance the stemness of liver cancer cells and contribute to sorafenib resistance. Although the Wnt/β-catenin pathway is well recognized for its role in HCC progression, the impact of APs on this pathway remains unclear. Further studies are essential to shed light on this area.

Through animal models and* in vitro* experiments, we revealed that APs effectively inhibited HCC malignancy via Wnt/β-catenin signaling. Astragalus polysaccharides (APs) also regulated the key epithelial–mesenchymal transition (EMT) markers, which are implicated in cancer progression, metastasis, drug resistance, and metabolic reprogramming. These results deepen our understanding of HCC pathogenesis and support the potential of APs as a therapeutic agent.

## Materials and Methods

### Cell Culture and Drug Processing

Human HuH7 (SNL-085) and HCCLM3 (SNL-166) cell lines were sourced from Wuhan Sunncell Technology Co., Ltd. (China). Human THLE-3 cells were purchased from ATCC (USA). These cells were cultivated in DMEM medium, which was added with 1% penicillin/streptomycin and 10% FBS (Gibco, USA) at 37°C and 5% CO_2_. The APs was purchased from Solarbio (China), and cells or nude mice were treated with APs according to the experimental protocol.

### CCK-8

HuH7, HCCLM3, and THLE-3 cells were seeded into 96-well plates overnight at 37°C with 5% CO_2_. They were treated with varying concentrations of APs (25-3200 µg/mL) for 48 hours. Following treatment, the cells were incubated with a 10% CCK-8 solution for 1 hour. Finally, the OD values were measured at a wavelength of 450 nm using a microplate reader to determine the half-maximal inhibitory concentration (IC_50_) of APs against HCC and normal liver epithelial cells.

### Western Blot

After treating HuH7 and HCCLM3 cells with the IC_50_ value of APs for 24 hours, the cells were rinsed twice with PBS. Cell lysates were prepared using RIPA buffer enriched with protease inhibitors (Sigma, USA). Proteins were separated by 10% SDS-PAGE and transferred onto PVDF membranes (Millipore, USA). Following a 1-hour block with 5% skim milk, membranes were incubated with primary anti-E-cadherin (Abcam, ab40772, UK), anti-N-cadherin (Abcam, ab76011, UK), anti-vimentin (Abcam, ab92547, UK), anti-β-catenin (Abcam, ab32572, UK), anti-p-β-catenin (Proteintech, 51067-2-AP, USA), anti-GSK3β (Proteintech, 51065-1-AP, USA), anti-p-GSK3β (Proteintech, 14850-1-AP, USA), and anti-GAPDH (Abcam, ab181602, UK) antibodies overnight at 4°C. With 3 rinses, membranes were incubated for 2 hours with an HRP-conjugated goat anti-rabbit IgG secondary antibody (Abcam, ab205718, UK). ECL kit (OMIGET, China) and chemiluminescent imaging system (Bio-Rad, USA) were used to visualize the protein bands.

### Invasion and Metastasis

Cells HuH7 and HCCLM3 were cultured onto the Transwell chambers (0.8 μm pore size, Corning, USA) pre-coated with Matrigel. The upper chamber cells were treated with the IC_50_ concentration of APs. The lower counterpart was coated with 10% FBS-containing culture medium, which served as a chemoattractant. The chambers were incubated at 37°C for 24 hours. Following incubation, cells in the upper chamber that failed to penetrate were removed using a cotton swab. Those in the lower chamber were fixed for 30 min using 4% formaldehyde and then stained with 0.1% crystal violet for 20 min. At the end of the experiment, we evaluated the invasion capacity of the cells under a microscope. For migration assays, Transwell chambers without Matrigel were used.

### Apoptosis Detection

To assess apoptosis, HuH7 and HCCLM3 cells were treated with the IC_50_ concentration of APs for 24 hours, followed by staining with FITC-conjugated Annexin V and PI using the Annexin V-FITC/PI Apoptosis Detection Kit (YEASEN, China). Cells were incubated in the dark at room temperature for 15 min and stabilized with binding buffer. Apoptosis levels were determined via a flow cytometer (Agilent, USA).

### Animal Experiments

Female BALB/c nude mice (4-6 weeks old, 18-20 g) were purchased from Hangzhou Ziyuan Laboratory Animal Technology Co., Ltd. and housed in an SPF facility with ad libitum access to food and water. After a 1-week acclimatization period, xenograft tumors were established by subcutaneously injecting 100 μL of HuH7 cell suspension (5 × 10^6^ cells) into the right flank. Mice were then randomly divided into 4 groups (n = 6): model control (NC, saline), low-dose APs (100 mg/kg), medium-dose APs (200 mg/kg), and high-dose APs (400 mg/kg). Twenty-four hours after injection, mice in the APs treatment group received intraperitoneal injections of APs at doses of 100, 200, and 400 mg/kg for treatment, while mice in the NC model group received an equal volume of intraperitoneal saline injection. The administration continued for 12 consecutive days. At the end of experiment, mice were humanely killed, and tumors were resected and weighed to quantify tumor growth. The animal experiments involved in this study were reviewed and approved by the Ethics Committee of Zhejiang Luoxi Medical Technology Co., Ltd., Hangzhou, China (Approval no: LX4824121301, Date: 2024-12-13). Patient consent was not required in accordance with local or national guidelines. The dosage of APs was determined based on the effective dose range reported by He et al.^17^

### Immunohistochemistry

Hepatocellular carcinoma (HCC) tumor tissue samples were fixed in 4% paraformaldehyde at 4°C overnight, and were embedded in paraffin and sectioned into 4 µm slices. After deparaffinization and rehydration using graded ethanol, the sections were treated with 3% hydrogen peroxide for 10 min to hinder endogenous peroxidase activity. Antigen retrieval was performed using EDTA buffer in a microwave for 20 min. The sections were blocked with normal goat serum at 37°C for 30 min to reduce nonspecific binding. Primary antibodies against Ki-67 (ab16667), E-cadherin (ab40772), N-cadherin (ab76011), and vimentin (ab92547) were applied and incubated at 37°C for 1.5 hours, all from Abcam, UK. After the addition of HRP-conjugated secondary antibody, the sections were incubated for another 30 min at the same temperature. The sections were visualized with DAB and counterstained with hematoxylin. Sections were dehydrated, cleared with xylene, and subsequently examined under a microscope to document the findings.

### Statistical Analysis

All experiments were performed independently ≥3 times, with results reported as the mean ± SD. GraphPad Prism 8.0 (GraphPad Software; San Diego, USA) was employed for data analysis. Significance between 2 groups was assessed using a *t*-test, while one-way ANOVA was conducted to evaluate significance among three or more groups. Significance was determined at *P* < .05.

## Results

### Astragalus Polysaccharides Inhibit Hepatocellular Carcinoma Progression in Mice

**Figure 1.**
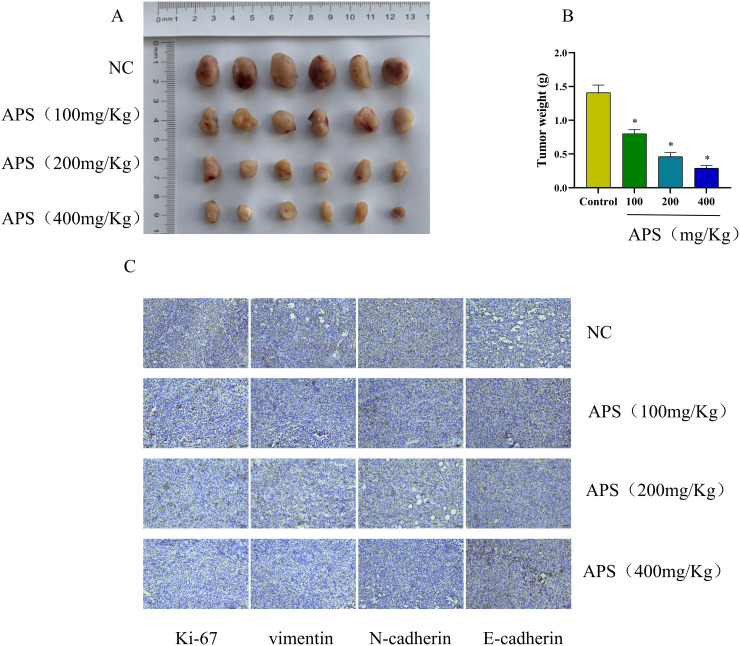
Astragalus polysaccharides (APs) inhibit HCC progression in mice. (A) Nude mice with xenograft tumors were treated with APs and were killed after 12 days. The tumor size was measured; (B) tumor weight; (C) the tissues of nude mice were prepared as paraffin sections, with IHC performed for evaluation of Ki-67 and epithelial–mesenchymal transition proteins.

In an effort to understand how APs affect liver cancer, we established a xenograft tumor model in mice and administered APs at doses of 100, 200, and 400 mg/kg. APs effectively hindered tumor growth in a dose-dependent manner (Figure 1A and B). According to IHC analysis (Figure 1C), as APs doses increased, Ki-67, vimentin, and N-cadherin expression declined, while E-cadherin levels contrastingly rose. Overall, these data revealed that APs can inhibit EMT and thus curb HCC progression.

### Astragalus Polysaccharides Attenuate the Proliferation, Migration, and Invasion of Hepatocellular Carcinoma Cells

**Figure 2.**
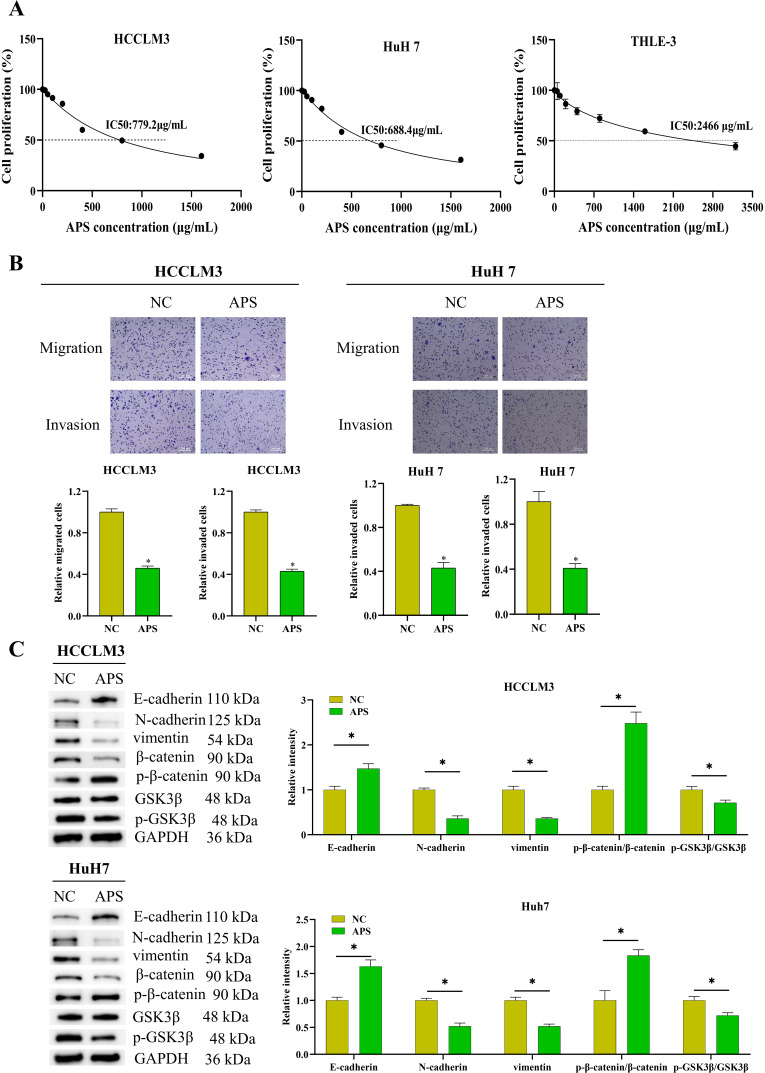
Astragalus polysaccharides (APs) attenuates the proliferation, migration, and invasion of hepatocellular carcinoma (HCC) cells. (A) HCCLM3, HuH7, and THLE-3 cells were treated with different concentrations of APs for 48 hours. The anti-cancer effects of APs were evaluated via CCK-8 assay, with the IC_50_ values determined; (B) cells were treated with the IC_50_ concentration of APs for 48 hours, followed by Transwell detection of cancer cell migration and invasion; (C) cells were treated with the IC_50_ concentration of APs for 48 hours, followed by Western blotting (WB) detection of epithelial–mesenchymal transition (EMT) and Wnt-related proteins (*P* < .05).

We treated HCCLM3 and HuH7 cells with APs and measured cell proliferation using the CCK-8 assay. The repressive effect of APs on cell proliferation was dose-dependent, with IC_50_ values of 779.2 µg/mL for HCCLM3 and 688.4 µg/mL for HuH7 cells. Meanwhile, when treating the normal liver cell line THLE-3 with APs, we observed an IC_50_ value as high as 2679 μg/mL, which was significantly higher than that in HCC cell lines. This indicated that APs exhibited selective toxicity toward HCC cells, causing less damage to normal liver cells at equivalent doses (Figure 2A). As revealed by Transwell assays, APs markedly hindered the migration and invasion of these cells (Figure 2B). Western blotting (WB) analysis showed that APs primarily inhibited the Wnt/β-catenin signaling pathway by downregulating β-catenin and p-GSK3β levels while increasing p-β-catenin levels. Additionally, APs treatment reduced Vimentin and N-cadherin levels and upregulated E-cadherin expression in cells (Figure 2C). These results suggested that APs may suppress the proliferation, migration, and invasion of HCC cells by modulating the Wnt/β-catenin signaling pathway and EMT-related proteins.

### Astragalus Polysaccharides Attenuate Hepatocellular Carcinoma Progression by Disrupting Wnt/β-catenin Signaling

**Figure 3.**
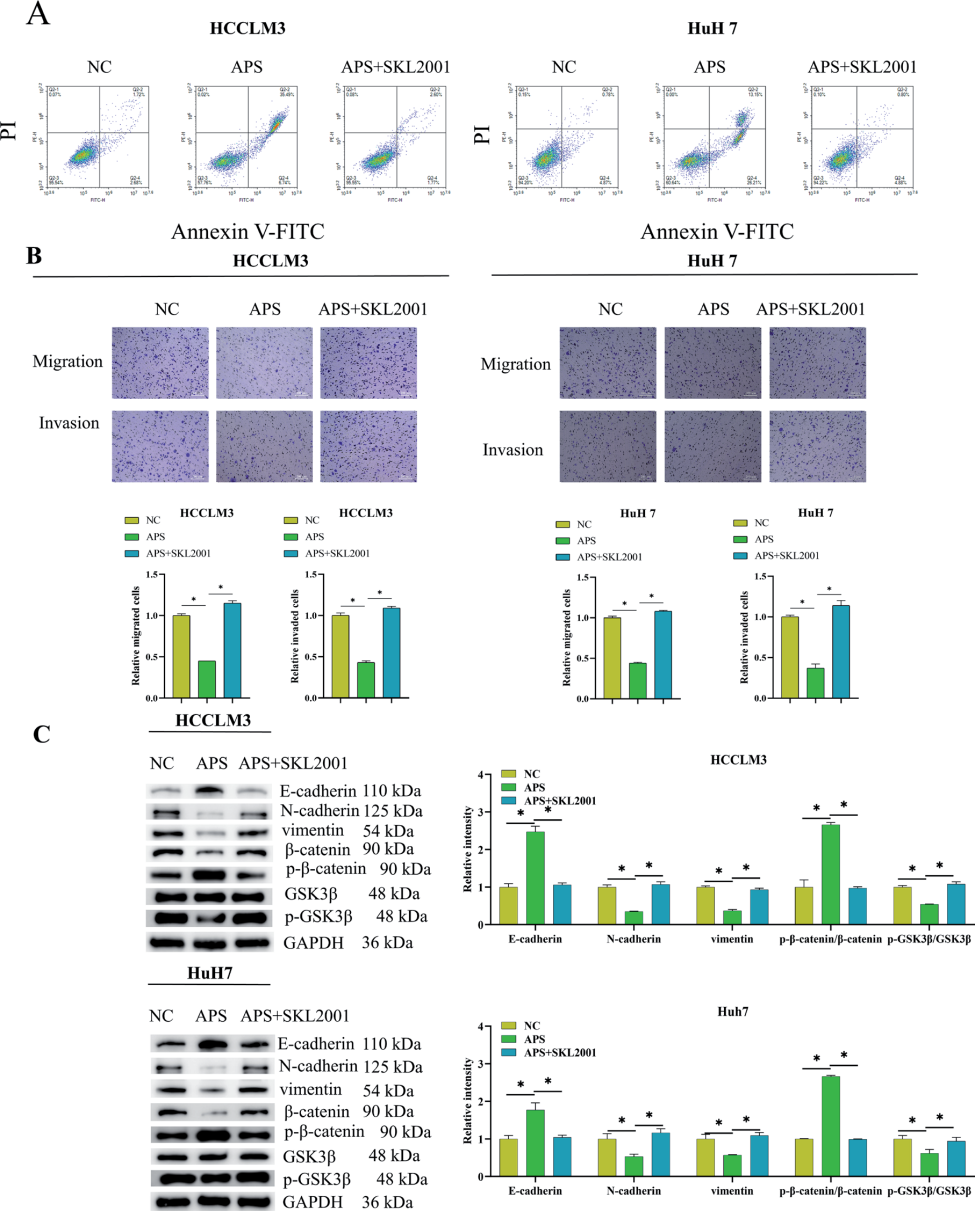
Astragalus polysaccharides (APs) attenuate hepatocellular carcinoma (HCC) progression by inhibiting the Wnt/β-catenin signaling pathway. (A) Cells were co-treated with APs at its IC_50_ concentration and 5 µM SKL2001 for 48 hours, followed by PI-ANNEXIN V flow cytometry for cancer cell viability; (B) after 48-hour co-treatment with the IC_50_ concentration of APs and 5 µM SKL2001, Western blotting (WB) was performed to analyze epithelial–mesenchymal transition (EMT) and Wnt-related proteins; (C) after 48-hour co-treatment with the IC_50_ concentration of APs and 5 µM SKL2001, Transwell assay was used for detecting cancer cell migration and invasion. SKL2001, as a Wnt/β-catenin pathway activator (*P* < .05).

We designed *in vitro* rescue experiments to determine whether APs inhibits HCC progression by targeting the Wnt/β-catenin pathway, which is critical in regulating cancer cell behaviors.^18^^,^^19^ Flow cytometry using PI-Annexin V revealed that APs clearly increased apoptosis in HCCLM3 and HuH7 cells, a trend that was reversed by the Wnt/β-catenin agonist SKL2001 (MCE, USA) (Figure 3A). Transwell confirmed the repressive impact of APs on migration and invasion of these cells, but these effects were restored by the Wnt/β-catenin agonist (Figure 3B). Western blotting (WB) analysis revealed that APs drastically reduced Wnt/β-catenin signaling. This treatment also manipulated EMT markers by inhibiting vimentin and N-cadherin expression and promoting E-cadherin. These changes were effectively reversed by a Wnt/β-catenin pathway agonist (Figure 3C). The data suggested that APs effectively impeded the malignant progression of HCC by repressing Wnt/β-catenin signaling and interfering with EMT.

## Discussion

HCC is a notoriously aggressive cancer, often leading to a grim prognosis for those affected. Although current treatments have had some impact, the overall survival rates of patients with HCC are still far from satisfactory,^20^^,^^21^ suggesting considerable room for improvement in therapeutic strategies. Our study has identified APs as a potential new therapeutic approach. We showed that APs can profoundly suppress HCC cell growth and spread by targeting Wnt/β-catenin signaling, thus interfering with the expression of key EMT markers. These findings not only provide new insights into HCC biology but also offer hope for patients facing this challenging cancer.

APs have shown remarkable biological and pharmacological properties, with therapeutic effects observed in several cancers.^22^^-^^24^ Notably, APs can induce breast cancer cells (MCF-7) to arrest in G1 phase and bladder cancer cells in G0/G1, effectively suppressing their proliferation.^6^^,^^25^ Despite these promising results, research on APs in HCC is still in its infancy, with only a few studies available. Li et al^2^ reported that APs could reduce O-GlcNAc acylation in HCC cells, thereby increasing ER stress and enhancing doxorubicin-induced apoptosis. Similarly, He et al^26^ showed that APs can counteract immunosuppression mediated by PD-L1 in HCC via miR-133a-3p/MSN. Both studies strengthened the reliability of our findings, solidifying that APs can effectively curb HCC progression. Our study, however, focused on a different pathway, and demonstrated that APs obstruct Wnt/β-catenin signaling in HCC cells, thereby attenuating cell proliferation, migration, and invasion. Evidence continues to accumulate that many polysaccharides show anti-tumor properties.^10^ As reported in earlier research, rose polysaccharides induce apoptosis in cervical cancer cells by targeting the PI3K/AKT/mTOR pathway.^27^ Meanwhile, *Ganoderma lucidum* polysaccharides promote M1 macrophage polarization and inhibit HCC growth via MAPK/NF-κB signaling.^28^ Despite these insights, the impact of APs on Wnt/β-catenin signaling in HCC remains unclear. Our study addresses this gap, by showing how APs can influences this pathway and laying the groundwork for future therapeutic innovations.

The Wnt/β-catenin pathway represents one of the most extensively studied oncogenic cascades, implicated in multiple cancer hallmarks.^29^ For instance, its aberrant activation drives proliferation, stemness, and metastasis in colorectal cancer,^30^^,^^31^ while its inhibition can reverse therapy resistance in breast and non-small cell lung cancers.^32^^-^^34^ Emerging evidence further suggests that modulating this pathway may remodel the tumor immune microenvironment and counteract immune evasion.^35^^,^^36^ Given these multifaceted roles, targeting Wnt/β-catenin has become a promising therapeutic strategy across malignancies. Given that dysregulation of Wnt/β-catenin signaling is a genetic alteration in HCC, targeting it has become a focus for developing new therapies.^11^ Pantothenate kinase 1, for instance, can inhibit HCC cell progression by negatively regulating this pathway.^37^ Likewise, TRIM36 has demonstrated its ability to suppress tumor development and promote apoptosis in HCC via Wnt/β-catenin signaling.^38^ Our study, building on prior research, demonstrated that repressing Wnt/β-catenin signaling substantially hinders HCC progression. A significant contribution of our study was the novel finding that ARS can inhibit this pathway, suggesting a new and promising option for HCC treatment.

Our study revealed that APs treatment can interfere with the EMT process in HCC. During EMT, epithelial tumor cells transition to a mesenchymal phenotype, gaining enhanced migratory and invasive capabilities.^39^ This shift is typically marked by lowered E-cadherin and heightened N-cadherin and Vimentin expression.^40^ We found that when HCC cells were treated with APs, E-cadherin expression increased while N-cadherin and Vimentin levels decreased, hinting at EMT inhibition. However, activating Wnt/β-catenin signaling disrupted these effects, suggesting that APs can interfere with EMT through this signaling pathway. This discovery is consistent with previous work by Yang et al,^41^ who reported that APs can hinder breast cancer development by targeting Wnt/β-catenin signaling, suggesting that APs may have a conserved mechanism across different cancers. However, although previous studies have reported the effects of APs on EMT in breast cancer, its specific role and mechanisms in HCC remain unclear. Our study not only confirmed the regulatory effect of APs on EMT in HCC but also elucidated its underlying molecular mechanisms, thereby partially filling this knowledge gap in the field.

The current study comprehensively evaluated the inhibitory effects of APs on HCC using both animal and in vitro cell models, elucidating its molecular mechanism in suppressing HCC cell proliferation, migration, and invasion by inhibiting the Wnt/β-catenin signaling pathway and modulation of EMT marker expression. These findings offer novel strategies and theoretical support for HCC treatment. However, the study had several limitations. Given the differences between animal models and human disease, as well as the complexity of prognosis prediction and treatment selection in HCC patients,^42^ the applicability of our findings to humans requires further validation. Additionally, while this study confirms the inhibitory effects of APs on HCC, the optimal therapeutic dosage and long-term safety profile remain to be determined, along with its feasibility and safety in clinical applications. Therefore, future research should focus on addressing these limitations to develop more effective strategies for HCC treatment.

## Data Availability

The data that support the findings of this study are available on request from the corresponding author.
